# The emerging role of mitochondria in the pharmacological and toxicological effects of *Tripterygium wilfordii* Hook F: functions, targets and new therapeutic applications

**DOI:** 10.1186/s13020-025-01170-6

**Published:** 2025-07-16

**Authors:** Zhonghao Liu, Dan Li, Xiongwen Yang, Xisha Chen, Chengxiao Fu

**Affiliations:** 1 The First Affiliated Hospital, Department of Pharmacy, Hengyang Medical School, University of South China, Hengyang, 421001 Hunan China; 2 The First Affiliated Hospital, Cancer Research Institute, Hengyang Medical School, University of South China, Hengyang, 421001 Hunan China; 3 Hunan Provincial Key Laboratory of Multi-Omics and Artificial Intelligence of Cardiovascular Diseases, University of South China, Hengyang, 421001 Hunan China; 4 School of Pharmaceutical Science, Hengyang Medical School, University of South China, Hengyang, 421001 Hunan China

**Keywords:** *Tripterygium wilfordii* Hook F, Celastrol, Triptolide, Mitochondria, Pharmacology, Toxicity

## Abstract

*Tripterygium wilfordii* Hook F (TWHF) is a traditional Chinese medicine with multifaceted pharmacological properties, has faced clinical application challenges due to its dose-limiting organ toxicity. Through a systematic analysis of current literature, this review deciphers the dual regulatory role of mitochondrial function in mediating both therapeutic efficacy and adverse effects of TWHF-derived active compounds. We elucidate that processes such as mitochondrial biogenesis, mitochondrial fusion and fission, mitophagy and mitochondrial apoptosis pathways influence the therapeutic efficacy and adverse effects of TWHF active ingredients. In addition, we review innovative dosage forms and derivatives of TWHF that exploit mitochondrial targeting to enhance their pharmacological efficacy. This article offers a concise overview pharmacological and toxicological effects of the principal active components of TWHF, in particular triptolide and celastrol, with a particular emphasis on elucidating the critical role of mitochondria in these processes. Further high-quality basic studies are essential to strengthen the link between mitochondrial function and the active compounds of TWHF, offering more diverse options for their clinical development.

**Graphical Abstract:**

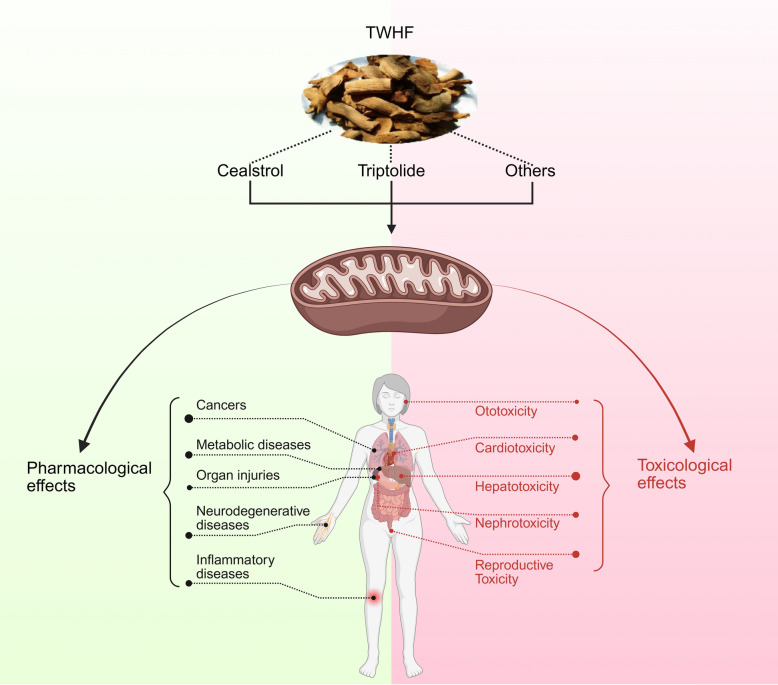

## Introduction

*Tripterygium wilfordii* Hook F (TWHF), commonly known as the “thunder god vine,” is widely distributed in South China, Korea and Japan. It was first recorded in the year 1476 as a traditional Chinese medicine [1]. Xylem of TWHF has been utilized in treating a broad spectrum of autoimmune disorders, inflammatory diseases, which boast a long-standing history of medicinal use. The pharmacological activity of TWHF derives from the presence of hundreds of active compounds, including sesquiterpenes, diterpenes, triterpenes, and so on [2]. However, these active compounds can also cause many serious toxicities such as hepatotoxicity, reproductive toxicity, nephrotoxicity, and cardiotoxicity [3]. Considerable research has focused on two major bioactive compounds, triptolide and celastrol, highlighting their broad-spectrum anti-inflammatory, anti-tumor, and other therapeutic effects [4, 5]. And even according to the important insights from the journal ‘Cell’, triptolide and celastrol are two of the five most promising natural compounds for future drug development [6].

Mitochondria, a crucial organelle in cell physiology, is known as the “power house” for its key roles in cellular energy production [7]. Mitochondria are involved in various processes such as ROS levels, calcium signaling, apoptosis, free radical generation and lipid metabolism, which are essential for cell survival and viability [8, 9]. More and more evidence shows that the occurrence and progression of cancer, neurodegeneration, and other diseases are closely linked to mitochondrial dysfunction [10, 11]. Additionally, due to the multifunctional nature of mitochondria, they are inevitably implicated in the toxic mechanisms of many drugs [12]. In the pharmacological and toxicological mechanisms of TWHF compounds, the dual regulatory role of mitochondrial function is particularly significant. On the one hand, TWHF components exert various therapeutic effects by targeting mitochondrial pathways to regulate ROS levels, modulate membrane potential, and maintain mitochondrial homeostasis, thereby triggering mechanisms such as mitophagy and mitochondrial apoptosis [13, 14]. On the other hand, mitochondrial dysfunction serves as a key mediator of the multi-organ toxicities induced by triptolide [15]. Without a doubt, a substantial amount of basic research is still needed in the future to clarify the mechanism underlies mitochondrial interaction with TWHF.

Recently, many articles have reviewed the pharmacological of the active compounds of TWHF directly from the perspective of diseases [16] or organs [17]. Increasing evidence highlights mitochondria as a central hub mediating both the therapeutic and toxic outcomes of TWHF compounds, largely due to their indispensable roles in energy metabolism, redox homeostasis, and cellular fate regulation—processes that are tightly interwoven with mitochondrial integrity and function. However, they are rarely mentioned in the peer reviewed literature. In this review, we will focus on mitochondria as a unique perspective to summarize the current knowledge about the pharmacological and toxicological effects of major compounds in TWHF. This perspective provides a unifying framework to reconcile the dual nature of TWHF and its compounds, paving the way for targeted interventions in inflammation, cancer, and beyond.

## The main active compounds of *Tripterygium wilfordii* Hook F

### Celastrol

**Fig. 1:**
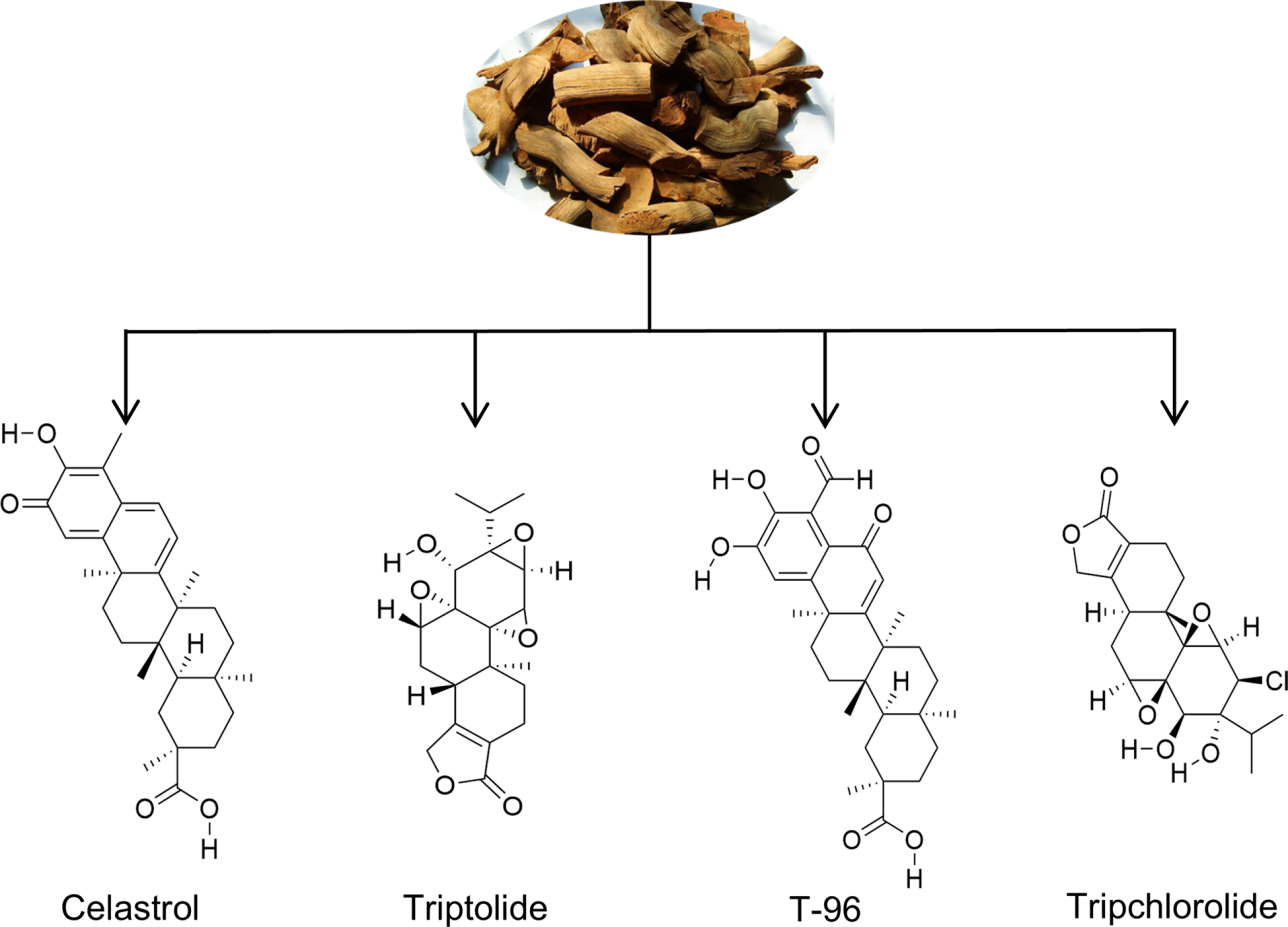
The main active ingredient of TWHF

Celastrol (C_29_H_38_O_4_, structured in Fig. 1), the primary quinone methylated triterpenoid compound found in TWHF, was initially isolated from TWHF in 1936 [18]. It is a red powder with a molecular weight of 450.610 g/mol [19]. After years of extensive research, celastrol has demonstrated potent pharmacological activities. Due to its remarkable pharmacological activities, celastrol is expected to be an excellent therapeutic agent to solve many public health problems. However, the clinical translation of celastrol has been impeded by its toxicity, limited water solubility, and low oral bioavailability. It has been demonstrated that the great pharmacological activity of celastrol are associated with the intramolecular hydrogen bonding formed by the carbonyl group at the 2-position and the enolic hydroxyl group at the 3-position.[20].

Researchers are expanding their focus from the chemical structure of celastrol to more comprehensive molecular mechanisms. This shift provides greater insight into the pharmacological activity of celastrol while also addressing potential toxicity and other challenges. So far, HSP90, NF-κB, ROS, Nur77, PI3K, MAPK, etc. are the mainly recognized molecular targets of celastrol [21]. These targets influence numerous signaling pathways and have demonstrated substantial therapeutic efficacy in inflammatory diseases, various solid tumors, metabolic disorders, Alzheimer’s disease and other medical conditions [21, 22]. It is noteworthy that many of these regulated molecules and signaling pathways have intimate connections with mitochondria [23].

### Triptolide

Triptolide (C_20_H_24_O_6_, structured in Fig. 1) was first isolated from TWHF in 1972 [24]. It is an epoxide diterpene lactone compound with a molecular weight of 360.401 g/mol, and has a significant inhibitory effect on certain inflammatory diseases and a variety of solid tumors [4]. For example, triptolide induces mitochondrial dysfunction, cell cycle arrest, oxidative stress, apoptosis and autophagy in a wide range of tumor cells through the regulation of key molecules such as HSP70, JAK2, NF-κB, Bcl-2/Bax, caspase-8, PI3K, MAPK, MPK1, ERK-1/2, JNK-1/2, Drp1, ROS and NO [25]. By regulating TNF-α, IFN-γ, RANTES, IL-1β, IL-8, IL-6, COX-2, AMPK, and NO, etc., triptolide can also play a protective role against various types of inflammation and its related diseases like rheumatoid arthritis, fatty liver disease, Parkinson's disease, and so on [26, 27].

The systemic toxicity of triptolide is more serious than that of celastrol. Triptolide brings about normal cell membrane impairment, mitochondrial dysfunction, oxidative stress, apoptosis, and autophagy, and subsequently induces damage to the liver, ovary, testis, kidney and heart [15]. Meanwhile, the structure of diterpene lactones contributes to the poor water solubility of triptolide, which limits its application in clinical settings [28].

### Others

Tripchlorolide and T-96 (structured in Fig. 1) are also derived from TWHF and exhibit structural similarities to triptolide and celastrol, respectively [29, 30]. Due to their similar structure, they can also exert certain pharmacological effects associated with mitochondria. A deeper understanding of the structure–activity relationship of the natural compounds of TWHF has led to an increasing number of related derivatives being synthesized by chemists. Derivatives such as CK21 [31] and (5R)-5-hydroxytriptolide [32] have been shown to have significant pharmacological activity. In addition to derivatives of exploration, the transformation of natural compounds is also a research focus. Against celastrol and triptolide, numerous nanoparticle delivery systems with high therapeutic efficacy and low toxicity are being developed [33–35]. To summarise the progress of basic research from a mitochondrial perspective is also of practical significance for exploring more possibilities of clinical application of triptolide.

## Mitochondria mediate the pharmacological effects of *Tripterygium wilfordii* Hook F active compounds

**Fig. 2:**
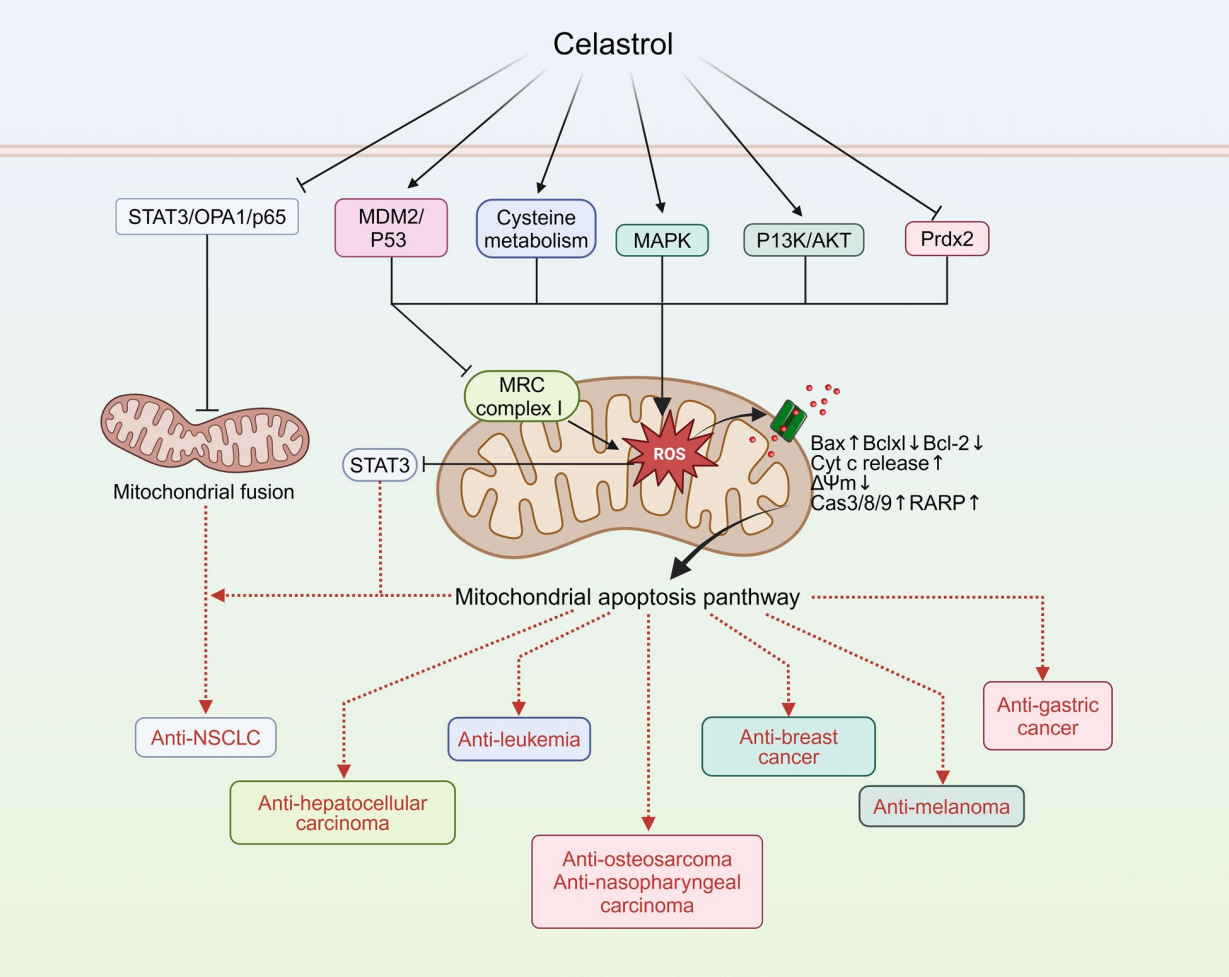
Celastrol exerts its anti-tumor effects via mitochondria-related pathways

**Fig. 3:**
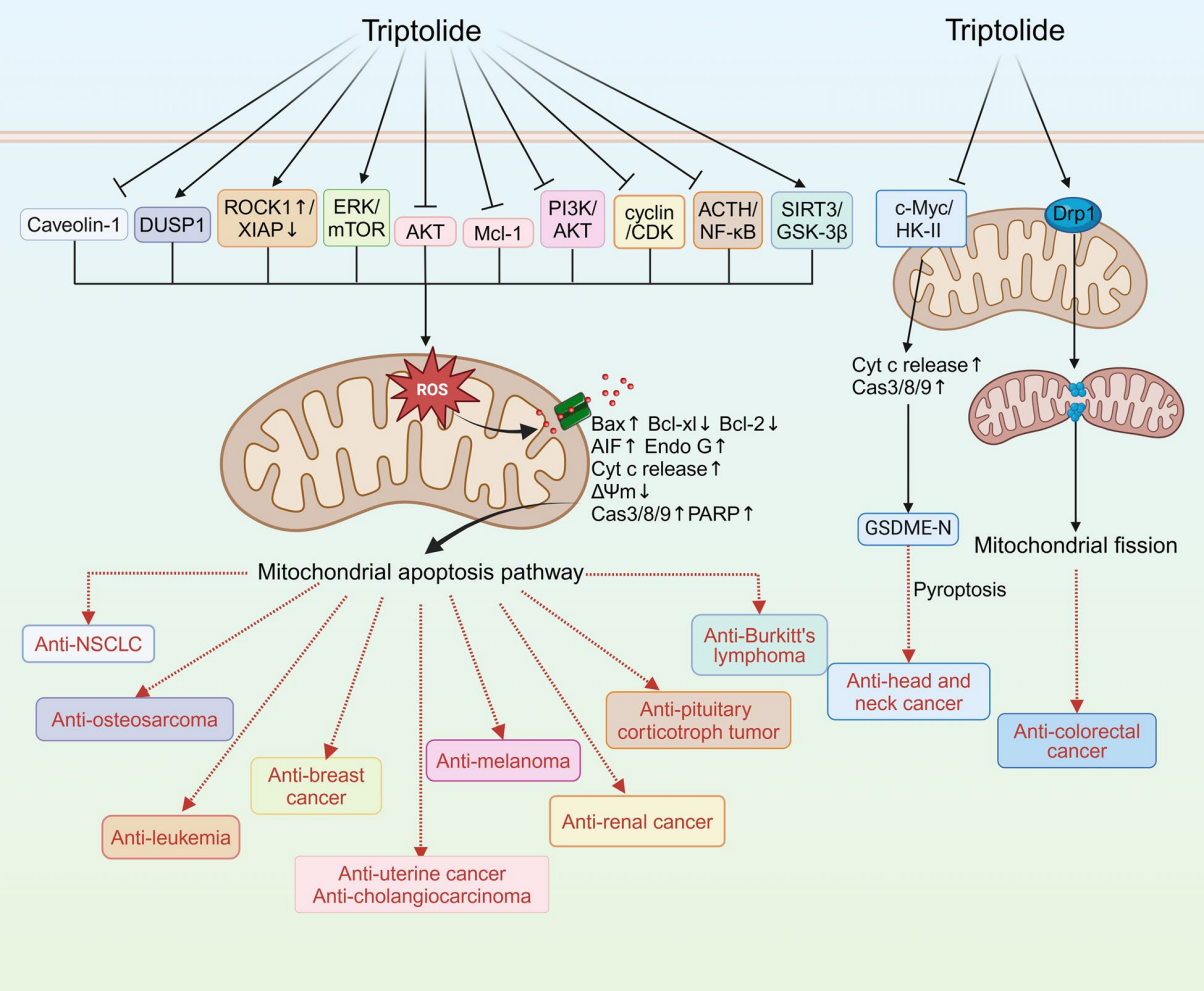
Triptolide exerts its anti-tumor effects via mitochondria-related pathways

**Fig. 4:**
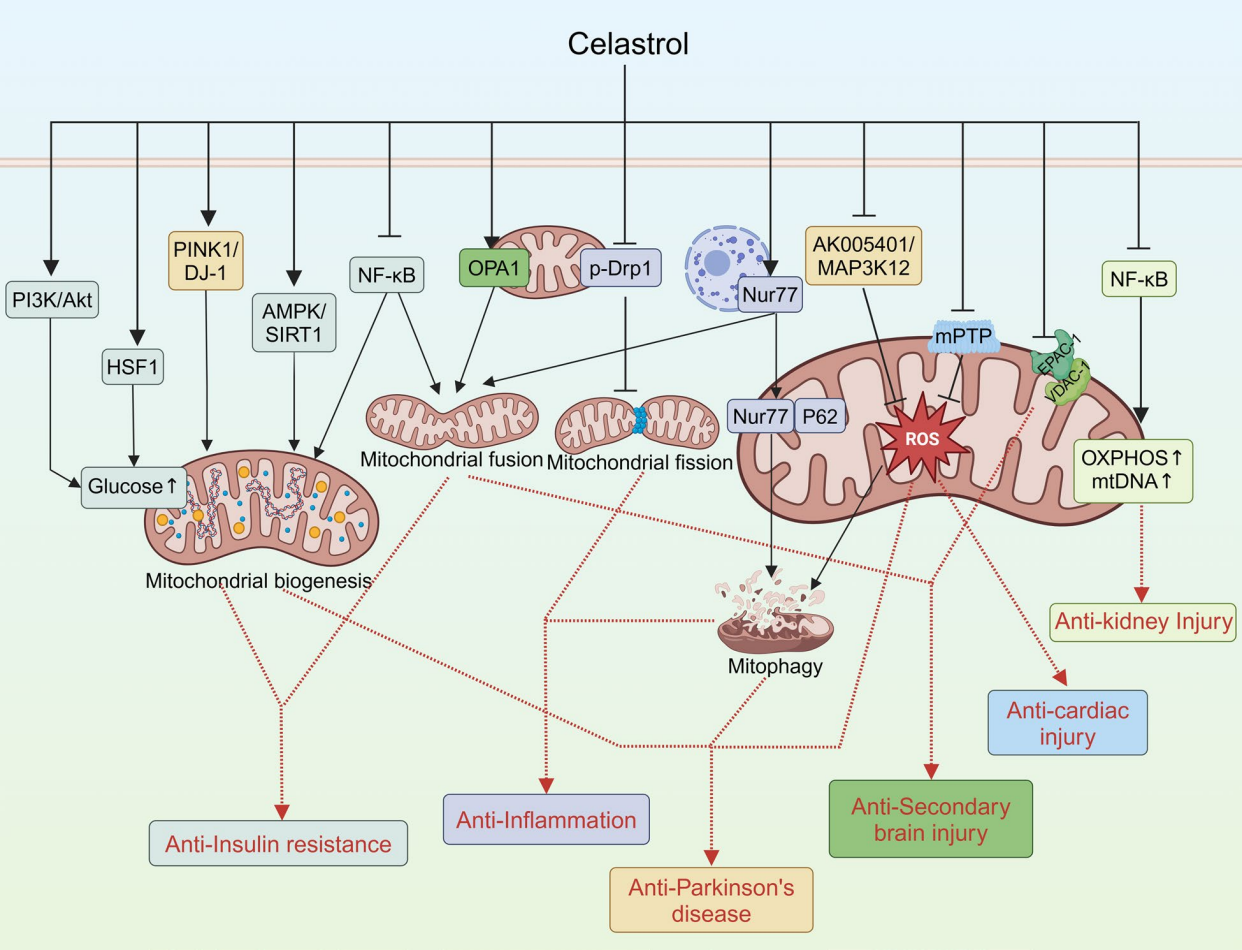
Celastrol exerts other pharmacological effects via mitochondria-related pathways

Fantastic advances have been made over the last 20 years in understanding of the mitochondrial apoptosis pathway and its role in cancer [36]. TWHF and its active compounds primarily regulate mitochondria-induced apoptosis of cancer cells through the following pathways: promoting mitochondrial apoptosis-related factors, including p53, the Bcl-2 family, Cytochrome C (Cyt c), and the caspase family [37]; inhibiting the expression of anti-apoptosis proteins associated with mitochondria [38, 39]; mediating oxidative stress and energy metabolism imbalance [40]; and regulating mitochondrial fusion and fragmentation [41]. The adjustment of mitochondria by the active compounds of TWHF in various tumor treatments is summarized in Table 1 and illustrated in Figs. 2 and 3. In addition to these pathways, celastrol also regulates mitochondrial biogenesis [42], mitochondrial glycolysis [43] and mitophagy [44], in a variety of cells. This allows it to exert a significant therapeutic effect on a wider range of diseases, including inflammatory, metabolic, neurodegenerative, cardiovascular, kidney, and brain injuries (Fig. 4).

**Table 1:** The active compounds of TWHF exert anti-tumor effects via mitochondria-related pathways

Type of cancer	Natural product	Experimental model	Dosage	Targets	Ref.
NSCLC	Celastrol	A549	0.5 μM, 2 μM, 4 μM	Cas-3/8/9 activation↑, cleaved PARP↑, Fas↑, FasL↑, ΔΨm↓, Bcl-2↓, Bax↑, Cyt c release↑,p-AKT↓	[45]
	Celastrol	H1650, H1975	0.5 μM, 1 μM, 2 μM, 4 μM	EGFR↓, ΔΨm↓, Bcl-2↓, Bax↑, Cas-3/7/8/9 activation↑, leaved PARP↑	[46]
	Celastrol	H460, PC-9, H520; Athymic BALB/c nude mice	1 μM, 2 μM, 4 μM; 2 mg/kg, 4 mg/kg	ROS↑, STAT3↓, ATF4↑, P-eIF2α↑, Bcl-2↓, Bax↑, Cas-3 activation↑,	[47]
	Celastrol	A549, HUVEC; BABL/c Nude mice	250 nM; 2 mg/kg	p-STAT3↓, OAP1↓, p-P65↑, NF-κB↑	[48]
	Triptolide	Lewis lung cancer cells	3 ng/ml, 5 ng/ml, 10 ng/ml, 40 ng/ml	ROS↑, mPTP↑, ATP production↑, ATP release↓	[51]
	Triptolide	NCI-H460, A549	50 nM, 100 nM	CAV-1↓, SIRT1↓, miR204-5p↑, p-AKT↓, p53↑, Bcl-2↓, Bax↑, Cas-3 activation↑, cleaved PARP↑	[52]
Hepatocellular rcinoma	Celastrol	H1299, HepG2	2 μM, 4 μM, 6 μM	HSP90↓, ROS↑, MRC complex I↑, EGFR↓, AKT↓, CDK4↓, JNK↑, Cas-3/9 activation↑	[56]
	Celastrol	HepG2	2 μM, 4 μM, 6 μM	AT2 receptor↓, Bcl-2↓, Bax↑, Cas-3 activation↑, ROS↑, eNOS↑, NO↑	[55]
	Celastrol	Male Sprague–Dawley rat	2 mg/kg, 4 mg/kg, 8 mg/kg	MDM2↓, p53↓, Bcl-2↓, Bcl-xl↓, Bax↑, Cyt c release↑, Cas-3/9activation ↑, cleaved PARP↑	[54]
Osteosarcoma	Celastrol	HOS, MG-63, U-2OS, Saos-2; Female BALB/c-nu mice	3 μM; 1 mg/kg, 2 mg/kg	ROS↑, p-JNK↑, Cas-3/8/9 activation↑, cleaved PARP↑	[58]
	Celastrol	MG-63, U-2OS, HOS	1 μM, 2.5 μM, 4 μM	Bcl-2↓, Bax↑, Cyt c release↑, Cas-3/8/9 activation↑	[59]
	Celastrol	HOS	3 μM	Bip↑, p-PERK↑, IRE1α↑, Calnexin↑, Ero1-Lα↑, PDI↑, CHOP↑, Bcl-2↓, Bax↑, Cyt c release↑, Cas-3/12 activation↑	[60]
	Celastrol	U-2OS	2.6 μM	GRP78↓, CHOP↑, Bcl-2↓, Bax↑, Cyt c release↑, Cas-3/8/9 activation↑, cleaved PARP↑	[61]
	Triptolide	U-2OS	200 nM	Fas↑, FasL↑, Bid↓, Bcl-2↓, Bax↑, Cyt c release↑, Cas-3/8/9 activation↑, cleaved PARP↑	[62]
	Triptolide	MG63, U-2OS, UMR-106; BALB/c nude mice	100 nM, 200 nM; 0.2 mg/kg	DUSP1↓, p-JNK1/2↑, p-ERK1/2↑, p-P38↑, Bcl-2↓, Bax↑, Cyt c release↑, Cas-3/9 activation↑	[63]
Leukaemia	Celastrol	HL-60, NB-4; Male BALB/c nude mice	0.5 μM; 2 mg/kg	L-cysteine↓, p53↑, Bax↑, Cas-3/9 activation↑	[65]
	Triptolide	U937, OCI-AML3, Jurkat, KG1, HL-60, K562	5 nM, 10 nM, 15 nM, 25 nM, 50 nM, 100 nM	XIAP↓, Mcl-1↓, Cas-3 activation↑, ΔΨm↓, Cyt c release↑, cleaved PARP↑	[66]
	Triptolide	HL-60	20 nM	p-JNK1/2↑, p-ERK↓, p-P38↓, Cyt c release↑, Cas-3/8/9 activation↑	[67]
	Triptolide	U937, Jurkat, HL-60	40 nM	ROCK1 activation↑,p-MLC↑, p-MYPT↑, Cyt c release↑, Cas-3/9 activation↑, cleaved PARP↑	[68]
	Triptolide	WEHI-3; Male BALB/c mice	60 nM; 0.2 mg/kg, 0.02 mg/kg	Ca2 + release↑, ΔΨm↓, Bax↑, ROS↑, Cas-3/8/9 activation↑, Fas↑, FasL↑, Cyt c release↑, Endo G↑, Apaf-1↑, AIF↓, ATF6a↓, TF6b↓, GRP78↓	[69]
Breast cancer	Celastrol	MCF-7	3 μM, 5 μM, 10 μM	Cyt c release↑, AIF release↑, Bcl-2↓, Bax↑, Cas-7/8/9 activation↑, Bid↓, cleaved PARP↑	[70]
	Celastrol	MCF-7, MDA-MB-231, T-549, MCF-10A	0.5 μM, 1 μM, 1.5 μM	ΔΨm↓, Bcl-2↓, Bax↑, ROS↑, Cas-3 activation↑, cleaved PARP↑, p-AKT↓, p70S6K1↓, 4E-BP1↓	[72]
	Triptolide	MCF-7	10 nM, 20 nM, 50 nM, 100 nM	Bax↑, Cas-3 activation↑, p62↓, Beclin-1↑, LC3B↑, p-ERK1/2↑, p-P38↑, p-mTOR↓	[75]
Cervical cancer	Triptolide	HeLa, Caski	20 nM, 50 nM, 100 nM	p-AKT↓, Mcl-1↓, ΔΨm↓, Cyt c release↑, Cas-3/8/9 activation↑, cleaved PARP↑	[76]
Endometrial cancer	Triptolide	HEC-1B	20 nM, 40 nM, 80 nM	Bcl-2↓, Cas-3/9 activation↑, cleaved PARP↑	[37]
Ovarian cancer	Tripchlorolide	CHO	20 ng/ml	Bax↑, Bcl-2↓, Cyt c release↑	[88]
Melanoma	Celastrol	B16; Male C57BL/6N mice	2 μM; 1 mg/kg, 3 mg/kg	ROS↑, Bax↑, Bcl-2↓, Cyt c release↑, AIF release↑, AKT↓, mTOR↓	[77]
	Triptolide	A375.S2	20 nM	p21↑, p27↑, cyclin A↓, Fas↑, FasL↑, CDC25A↓, Bcl-xl↓, Bax↑, AIF↑, ROS↑, NO↑, Ca2+ release↑, ΔΨm↓, Cas-3/8/9 activation↑	[78]
Myeloma	Triptolide	HS-sultan, IM9, RPMI8226, U266	25 nM	Mcl-1↓, ∆Ψm↓, Cyt c release↑, Smac/DIABLO release↑, Cas-3/9 activation↑	[79]
Gastric tumor	Celastrol	SGC-7901, BGC-823; Athymic BALB/c female mice	3 µM; 1.5 mg/kg	ROS↑, Prdx2↓, ATF4↑, p-eIF2α↑, p-PERK↑, CHOP↑, Cas-3 activation↑	[14]
	Tripchlorolide	AGS	20 ng/ml	pRB↓, Bax↑, Bcl-2↓, Cyt c release↑	[89]
Adrenal cancer	Triptolide	NCI-H295	125 nM	ROS↑, ΔΨm↓, Cyt c release↑, Apaf-1↑, AIF↑, Endo G↑, Cas-3/9 activation↑	[81]
Renal cell carcinoma	Triptolide	786-O, OS-RC-2	12.5 nM, 25 nM, 50 nM	Bax↑, Bcl-2↓, Bcl-xl↓, Cyt c release↑, Cas-3 activation↑, cyclin A↓, cyclin B↓, CDK1↓, CDK2↓	[82]
Cholangio carcinoma	Triptolide	HuCCT1, QBC939	25 nM, 50 nM, 100 nM	Mcl-1↓, Cas-3/7/9 activation↑, cleaved PARP↑	[83]
Nasopharyngeal carcinoma	Celastrol	HONE-1, NPC-039	1 μM, 2 μM, 4 μM	ΔΨm↓,Fas↓,FADD↑,TRADD↑,Fas↑,p-P38↓,p-ERK1/2↓, p-JNK1/2↑,TNF-1A/10B↑,Cas-3/8/9 activation↑	[80]
Pituitary corticotroph tumor	Triptolide	AtT20, D16v-F2; Female athymic mice	50 nM, 100 nM; 0.15 mg/kg	ACTH↓, NF-κB↓, p-ERK1/2↓, Cas-3 activation↑, Bax↑, Bcl-2↓	[84]
Burkitt's lymphoma	Triptolide	Raji, NAMALWA, Daudi; Male NOD/SCID mice	20 nM, 40 nM, 80 nM; 0.04 mg/kg, 0.12 mg/kg, 0.36 mg/kg	SIRT3↑, p-GSK-3β↑, Bax↑, Bcl-2↓, Cyt c release↑, Cas-3 activation↑	[38]
Head and neck cancer	Triptolide	HK1, C666–1, FaDu; BALB/c nude mice	50 nM; 0.1 mg/kg	c-Myc↓, HK-II↓, Bax↑, BaD↑, Cas-3 activation↑, cleaved GSDME↑	[87]
Colorectal carcinoma	Triptolide	DLD1, HCT-116	100 nM	ΔΨm↓, MRC complex I, Drp1↑	[86]

### Cancer

#### Lung cancer: mechanisms of celastrol and triptolide

Celastrol can induce apoptosis of human NSCLC cells by activating mitochondria and FasL-mediated pathways. It is characterized by activation of the caspase family, cleavage of PARP protein, reduction of mitochondrial membrane potential (Δψm), release of Cyt c, and reduction of Bcl-2/Bax ratio [45]. The same phenomenon was observed in gefitinib-resistant NSCLC cells. This suggests that by inducing apoptosis via the mitochondrial pathway, celastrol may be an effective strategy for the treatment of gefitinib-resistant NSCLC [46, 47]. Celastrol disrupts mitochondrial function and morphology by inhibiting OPA1, promotes apoptosis and inhibits angiogenesis in NSCLC cells [48]. The combined treatment of erastin and celastrol significantly promoted NSCLC cell death by activating the ROS-mitochondrial fission-mitophagy signaling pathway. The findings indicate that, in addition to the mitochondrial apoptosis pathway, exploring mitophagy as a strategy for celastrol treatment of NSCLC is warranted [49]. Triptolide can effectively inhibit the proliferation and metastasis of NSCLC [50]. After treating Lewis lung cancer cells with different doses of triptolide, molecular probes detected the impairment of various mitochondrial functions, indicating that triptolide may inhibit lung tumor growth through mitochondrial dysfunction [51]. Triptolide treatment of A549 and H460 cells resulted in a decrease in Caveolin-1 expression, which eventually led to the activation of AKT/BCL-2 induced mitochondrial apoptosis pathway [52].

#### Hepatocarcinoma: mechanisms of celastrol

The growth and apoptosis of Bel-7402 cells can be inhibited by celastrol in a dose- and time-dependent manner. The underlying mechanism may be related to the activation of the mitochondrial pathway [53]. Celastrol was demonstrated to suppress mitochondrial respiration and promote the accumulation of ROS in HCC mouse models and cellular systems, potentially through mechanisms involving inhibition of MMP-2 and MMP-9 [54], as well as suppression of MRC complex I activity [55]. ROS accumulation also induces HSP90 downregulation and activates the mitochondrial apoptosis pathway [56]. In addition, Celastrol selectively targets mitochondrial VDAC2, disrupting mitochondrial function, ultimately inducing ferroptosis in hepatocellular carcinoma [57].

#### Osteosarcoma: mechanisms of celastrol and triptolide

A variety of cell models and animal models have verified that celastrol play an anti-osteosarcoma role by promoting cell apoptosis through the mitochondrial pathway [58, 59]. Celastrol initially induces endoplasmic reticulum stress, leading to the up-regulation of endoplasmic reticulum stress transcription factors CHOP. This activation subsequently triggers the caspase family and reduces the Bcl-2/Bax ratio, ultimately initiating the mitochondrial apoptosis pathway [60, 61]. In U-2OS cells, triptolide induces apoptosis through the activation of death receptors and mitochondrial pathways [62]. Triptolide inhibits DUSP1 expression by reducing its promoter activity, resulting in the sustained activation of the three MAPK subfamilies, including ERK1/2, JNK1/2, and p38. Their sustained activation triggers the mitochondrial apoptotic pathway to exert anti-osteosarcoma effects [63].

#### Leukemia: mechanisms of celastrol and triptolide

Differentially regulated metabolites analyzed by metabolomics can probe the pathogenesis of diseases. Many of these metabolites are involved in mitochondrial metabolism and have an impact on mitochondrial function [64]. In combination with metabolomics, researchers have found that cysteine metabolism/ROS/p53 pathway provides a potential evidence of celastrol in the treatment of acute myeloid leukemia [65]. Triptolide induces mitochondrial dysfunction through reduces XIAP expression in leukemic cells. [66]. Another study proved triptolide-induced mitochondrial pathway is also independent of p53 in HL-60 cells [67]. The activation of ROCK1 and caspase-3 forms a positive feedback loop, which may account for the apoptosis in HL-60 cells induced by triptolide [68]. Furthermore, the release of Ca^2+^ resulting from mitochondrial dysfunction contributes to the anti-leukemic effects of triptolide [69].

#### Breast cancer: mechanisms of celastrol and triptolide

Celastrol treatment leads to the release of Cyt c and AIF from mitochondria, inducing apoptosis in human breast cancer cells [70]. Triple-negative breast cancer (TNBC), a distinct subtype of breast cancer, is characterized by its high invasiveness, propensity for metastasis, tendency for relapse, and unfavorable prognosis [71]. Shrivastava et al. found that celastrol induces apoptosis in TNBC cells by inhibiting the PI3K/AKT pathway and elevating mitochondrial oxidative stress [72]. Furthermore, the combination of celastrol with tamoxifen can effectively enhance the treatment efficacy in TNBC [73]. Yoon et al. found that after celastrol treatment, Ca^2+^ accumulated from the endoplasmic reticulum to mitochondria, causing dilation and vacuolization of mitochondria [74]. Therefore, celastrol may directly induce mitochondrial dysfunction to participate in the death of various breast cancer cells by causing Ca^2+^ homeostasis imbalance. In MCF-7 cells, triptolide may induce mitochondrial apoptosis and autophagy through the mTOR/ERK/p38 signaling pathway [75], suggesting that triptolide can also play an anti-breast cancer role consistent with celastrol.

#### Uterine cancer: mechanisms of triptolide

In HeLa cells AND HEC-1B cells, Triptolide can induce the classical mitochondrial apoptosis pathway by inhibiting Mcl-1 and AKT [76] and p53-independent signaling pathways [37], respectively. This indicates that triptolide has the same therapeutic potential for cervical cancer and endometrial cancer. More interestingly, these two studies have found that triptolide triggers the mitochondrial pathway but does not affect the expression of Bax. Therefore, the role of mitochondria in triptolide-induced apoptosis in human endometrial cancer cells warrants further exploration.

#### Melanoma: mechanisms of celastrol and triptolide

Celastrol and triptolide are involved in melanoma therapy through similar mechanisms. More specifically, the administration of celastrol has been demonstrated to effectively inhibit tumor growth in melanoma. This is achieved by triggering ROS-mediated caspase-dependent and -independent pathways, by inhibiting the PI3K/AKT signaling pathway [77]. In A375.S2 cells, triptolide induces apoptosis through Fas- and mitochondrial-mediated pathways [78]. Another study points out that triptolide-induced apoptosis of myeloma cells may also be related to the decreased transcription levels of Mcl-1 [79].

#### Other cancers: mechanisms of celastrol and triptolide

Induction of the mitochondrial pathway is pivotal for the broad anti-tumor activity of celastrol and triptolide. In addition to the cancers discussed above, celastrol induces apoptosis in nasopharyngeal cancer [80] and gastric cancer [14] via the mitochondrial pathway, involving crucial molecular targets such as MAPK and Prdx2, respectively. Triptolide has also exerted effects on the mitochondrial apoptosis pathway in various cancer including adrenal cancer [81], renal cell carcinoma [82], cholangiocarcinoma [83], pituitary corticotrophic tumor [84], Burkitt's lymphoma [38] via key molecular targets include Apaf-1, AIF, Endo G, cyclin/CDK, Mcl-1, ACTH, NF-κB and SIRT3/GSK-3β. In addition to mitochondrial related apoptosis pathway, recent research has also focused on the effects of triptolide on other mitochondrial functions, such as mitochondrial fusion-fission dynamics and pyroptosis. Mitochondrial fusion-fission dynamics play a pivotal role in the pathogenesis of colorectal tumor [85]. Triptolide reduces MRC complex I activity and affects mitochondrial division in the colorectal cancer cells [86]. In another study, triptolide inhibited the expression of c-Myc and mitochondrial HK-II in head and neck cancer cells, which activated the BAD/BAX-caspase-3 cascade reaction and cleaved GSDME by active caspase-3, ultimately leading to pyroptosis [87]. It has been reported that tripchlorolide, another component of TWHF, promotes apoptosis of ovarian and gastric cancer cells through promoting p53-independent pRB degradation and disrupting mitochondrial function [88, 89].

### Metabolic diseases

The prevalence of metabolic diseases has increased significantly over the past two decades, particularly obesity and T2DM, posing a global public health challenge [90]. Insulin resistance and obesity are mainly characteristics of T2DM [91]. Insulin resistance elevates insulin levels and promotes adipocyte lipid accumulation, resulting in obesity, thereby heightening the susceptibility to T2DM. Meanwhile, Obesity also disrupts cellular response to insulin, contributing to insulin resistance [92]. Metabolic studies have shown that mitochondrial dysfunction is present in individuals with obesity, insulin resistance and T2DM compared to healthy controls [93, 94]. Mitochondrial dysfunction leads to increased release of ROS and lipid metabolites and decreased β-oxidation and ATP production, affecting insulin secretion, signaling and function, further exacerbating insulin resistance [95].

It is well established that the NF-κB pathway plays a part in the development of insulin resistance and chronic inflammatory processes [96]. Celastrol reduced NF-κB expression in 3T3-L1 adipocytes, thereby enhancing mitochondrial fusion-fission dynamics. This created a positive feedback loop characterized by sequential events: mitochondrial repair attenuates oxidative stress, which diminishes inflammation and ultimately restores metabolic homeostasis [97]. In human skeletal muscle cells, celastrol also significantly increased mitochondrial activity via PI3K/AKT pathways against insulin resistance induced by mitochondrial dysfunction [98]. In C2C12 cells with palmitate-mediated insulin resistance, celastrol improved mitochondrial function by increasing the level of TCA cycle intermediates [99]. Mitochondrial biogenesis influences mitochondrial function and is also involved in the pathogenesis of insulin resistance [100]. Other studies reported that celastrol alleviates insulin resistance in rats by improving mitochondrial biogenesis and reducing oxidative stress. The underlying molecular mechanisms involve the upregulation of AMPK/SIRT1 and genes related to mitochondrial biogenesis [42, 101]. Furthermore, celastrol enhances muscle mitochondrial glucose uptake by activating HSF1 [102]. The above studies suggest that celastrol significantly improves mitochondrial function through multiple mechanisms and thereby restores normal glucose uptake and homeostasis. Improving energy metabolism exhibits potential for the treatment of metabolic diseases represented by obesity. The significant improvement in energy metabolism demonstrates the therapeutic potential of celastrol for various metabolic diseases. Beyond metabolic regulation, celastrol has also been shown to induce apoptosis in 3T3-L1 preadipocytes via a mitochondria mediated pathway [103]. Consistent with celastrol, triptolide has a broad range of pro-apoptosis activities via the mitochondrial pathways. Therefore, the potential mechanism by which triptolide induces mitochondrial apoptosis in adipocytes deserves attention.

### Inflammatory diseases

Mitochondria play a pivotal role in innate immunity and chronic inflammation. The maintenance of optimal mitochondrial homeostasis has emerged as an efficacious therapeutic strategy for the treatment of numerous chronic inflammatory conditions [104]. Mitophagy is an important form of autophagy. It is used to selectively remove dysfunctional or damaged mitochondria to maintain cellular homeostasis [105]. A growing body of research has demonstrated that autophagy represents an effective treatment for a number of chronic inflammatory diseases [106, 107]. Nur77 plays a pivotal role in a multitude of cellular processes as a member of the orphan nuclear receptor family [108–110]. Recent research focuses on the critical role of Nur77 in inflammation [109]. Hu et al. found that Nur77 is a key target of celastrol. Celastrol induces inflammatory mitochondrial autophagy in a Nur77-dependent manner to reduce liver inflammation [44]. Following celastrol treatment, Nur77 translocates from the nucleus to the mitochondria and binds to TRAF2 for ubiquitination [44]. Ubiquitinated Nur77 interacts with p62/SQSTM1 to form membrane-less condensates that sequester damaged mitochondria, leading to autophagy [13]. Interestingly, Nur77 can also target mitochondria to participate in apoptosis [111]. This will allow us to focus on the role of Nur77 in the celastrol-induced mitochondrial apoptosis pathway. Furthermore, celastrol was observed to inhibit mitochondrial division and suppress LPS-induced inflammatory responses by promoting phosphorylation of Drp1 at Ser637 site [112]. Moreover, in the context of acute pancreatitis, celastrol was demonstrated to exert a protective effect by inhibiting mitochondrial ROS production and the phosphorylation of RIPK1, RIPK3, and MLKL [113]. Due to its structural similarity to celastrol, T-96 also targets MRC complex I and inhibits mitochondrial ROS production, thereby attenuating oxidative stress in renal fibrosis [30].

### Neurodegenerative disease

Mitochondrial dysfunction, especially impairment of mitochondrial autophagy, is an important mechanism in the pathogenesis of Parkinson's disease [114]. Celastrol increased PINK1 and DJ-1 expression, activated mitochondrial autophagy and improved biogenesis, thus inhibiting dopaminergic neuronal apoptosis and exerting neuroprotective effects [115, 116]. In another study, celastrol improved mitochondrial function by down-regulating the AK005401/MAP3K12 pathway, which ultimately effectively reversed memory impairment [117]. In conclusion, celastrol has the potential to become one of the potential therapeutic agents for Parkinson's disease in the future.

### Secondary brain injury

Celastrol has emerged as a promising therapeutic agent in mitigating secondary brain injury induced by intracerebral hemorrhage. It localized to neuronal mitochondria to bind with EPAC-1 and antagonized the binding of EPAC-1 to VDAC-1, which ameliorated neuronal mitochondrial damage and consequently exerted a protective effect against damage caused by intracerebral hemorrhage [118]. In neuronal ischemia–reperfusion injury, expression of the mitochondrial fusion protein OPA1 is reduced, exacerbating neuronal mitochondrial disruption and injury [119]. Nevertheless, a recent study demonstrated that celastrol was able to reverse the observed decrease in OPA1 expression and attenuate mitochondrial morphological damage [120]. These evidence suggests a potential therapeutic role for celastrol in intracerebral hemorrhage induced Secondary brain injury.

### Other diseases

In contrast to its effects on tumour cells, celastrol has been demonstrated to reduce mitochondrial permeability transition pore (mPTP) opening [121] and inhibit mitochondrial ROS levels [122] in cardiomyocytes. Increased mitochondrial viability may allow celastrol to exert a protective effect against cardiovascular injury. Furthermore, celastrol has been demonstrated to possess a protective effect against kidney injury [123]. Following cisplatin-induced nephrotoxicity, celastrol exerted anti-apoptotic effects by inhibiting NF-κB activity and improving mitochondrial function [23].

## The involvement of mitochondria in the toxicological effects of *Tripterygium wilfordii* Hook F active compounds

**Fig. 5:**
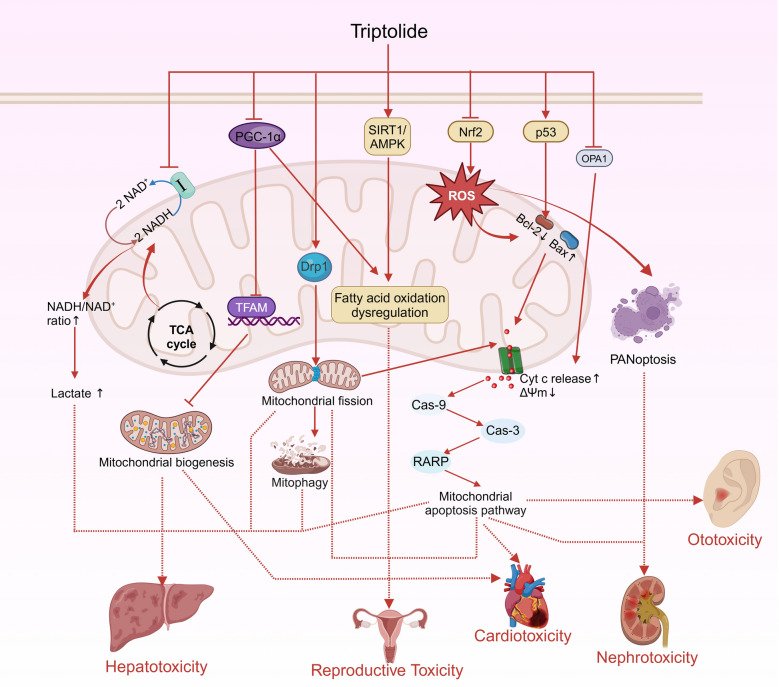
Triptolide produces toxicity through mitochondria-related pathways

As early as the sixteenth century, the Compendium of Materia Medica indicated that TWHF was toxic to humans. Currently, there has been a significant increase in reports on the toxic mechanisms of the main active ingredients of TWHF, particularly triptolide. Its toxicity encompasses hepatotoxicity, reproductive toxicity, nephrotoxicity, cardiotoxicity, ototoxicity, and so on. This has greatly restricted the clinical application of the active ingredients of TWHF. As shown in Table 2 and Fig. 5, Mitochondria are involved in the mechanism of triptolide toxicity. A focus on alterations in mitochondrial homeostasis may facilitate a comprehensive understanding of the underlying mechanisms of triptolide toxicity.

**Table 2:** Triptolide induces toxicity via mitochondria-related pathways

Toxicity	Experimental model	Dosage	Outcome	Ref.
Hepatotoxicity	Female Sprague–Dawley rats	200 μg/kg, 400 μg/kg	Inhibition of mitochondrial respiratory	[124]
	Female Wistar rats	400 μg/kg	Drp1-associated Mitochondria-dependent apoptosis	[127]
	Female Wistar rats	400 μg/kg	Drp1-associated mitochondrial dysfunction and mitophagy	[41]
	HepaRG Cell	100 nM, 200 nM, 300 nM	Induced apoptosis through Fas death and mitochondrial pathways	[125]
	Female Sprague–Dawley rats	0.1 μmol/L, 0.5 μmol/L	Induced mitochondrial swelling, mitochondrial membrane depolarization and activated mitochondrial permeability transition	[126]
Hepatotoxicity/Nephrotoxicity	C57BL/6 J mice	1 mg/kg, 2 mg/kg	Induced mitochondrial dysfunction leading to PANoptosis	[135]
Nephrotoxicity	BALB/c mice	0.2 mg/kg, 0.4 mg/kg	Induced mitochondrial DNA leakage and activates the cGAS-STING signaling pathway, leading to oxidative stress	[136]
Testicular toxicity	Male ICR mice	120 μg/kg	Mitochondrial dysfunctions induced by oxidative stress	[162]
	Male Sprague–Dawley rats	100 μg/kg	Disrupted mitochondrial morphology, mitochondrial membrane potential, and blood-testis barrier integrity	[167]
	Male ICR mice	0.05 mg/kg, 0.1 mg/kg	Induced mitochondrial fatty acid oxidation dysregulation by increasing AMPK phosphorylation	[128]
	Sprague–Dawley rats	400 μg/kg	Drp1-associated Mitochondria-dependent apoptosis	[130]
	Male ICR mice	30 μg/kg, 60 μg/kg, 120 μg/kg	ROS/JNK signaling pathway and Nrf2 signaling pathway associated-Mitochondria-dependent apoptosis	[129]
	Female Kunming mice	30 μg/kg, 60 μg/kg, 90 μg/kg	Disrupted mitochondrial morphology and function, and affects mitochondrial distribution in mouse oocytes	[131]
Cardiotoxicity	Male BALB/C mice	1.2 mg/kg	Nrf2-associated Mitochondria-dependent apoptosis	[40]
	Male C57BL/6 p53^−/−^ mice	1.2 mg/kg	Induced p53-dependent mitochondria-pathway	[133]
	H9c2 cells	80 nM	Mitochondrial dysfunctions induced by oxidative stress	[161]
	Male BALB/c mice	1.5 mg/kg	Depletion of mitochondrial mass and mitochondrial DNA copy number, disorders of mitochondrial membrane potential and mitochondrial oxidative phosphorylation	[159]
Ototoxicity	The inner ear stem cells	500 nM	Induced cleavage of OPA-1 impairs mitochondrial function	[137]

### Hepatotoxicity

The hepatotoxicity of triptolide is strongly associated with mitochondrial function and involves several biological processes controlled by mitochondria, including mitochondrial respiration, mitochondrial biogenesis, mitochondrial fission, mitophagy and mitochondrial apoptosis pathway (Fig. 5). Triptolide interferes with the reoxidation of NADH to NAD^+^ in the mitochondrial respiratory chain, and the imbalance in the NADH/NAD^+^ ratio leads to increased accumulation of lactic acid, which ultimately leads to liver injury [124]. The imbalance in the respiratory chain led to accumulation of mitochondrial ROS, which in turn triggered the mitochondrial pathway to induce apoptosis in liver cells [125, 126]. Drp1, which controls mitochondrial division, is also an important regulator of mitochondrial function and exerts an influential role in triptolide-induced hepatotoxicity. Triptolide treatment results in the recruitment of Drp1 to the outer mitochondrial membrane, which in turn leads to mitochondrial fission and mitophagy [41]. Excessive mitochondrial division leads to a massive release of Cyt c into the cytoplasm, further increasing apoptosis in L-02 cells [127]. These mechanisms ultimately point to severe hepatotoxicity.

### Reproductive toxicity

The reproductive toxicity of triptolide has been a significant concern, manifesting in reduced sperm counts, reduced viability, and ovarian cell damage. In TM4 cells and male rats, triptolide has been shown to impair mitochondrial function through the SIRT1/AMPK pathway, leading to disturbances in energy metabolism [128]. This process is further exacerbated by the suppression of PGC-1α expression [128]. Beyond its effects on energy metabolism, triptolide may exert direct pro-apoptotic effects on Sertoli cells [129]. It potentially through its significant upregulation of Drp1 expression in testicular interstitial tissue. This molecular alteration appears to disrupt the critical balance between mitochondrial fusion and fission processes, suggesting a mechanism involving impaired mitochondrial dynamics in triptolide-induced testicular toxicity [130]. The latest study found that triptolide promotes K63-linked GPX4 polyubiquitination and degradation, disrupting mitochondrial metabolism. In female mice, triptolide significantly affected mitochondrial function and morphology and impaired oocyte quality [131]. The specific molecular mechanisms may require further exploration in the future.

### Cardiotoxicity

Nrf1 and Nrf2 are members of the CNC-bZIP family, which together regulate mitochondrial oxidative homeostasis [132]. Triptolide stimulates the ROS-mitochondrial pathway by inhibiting Nrf2, leading to oxidative stress in myocardial tissue [40]. Finally, triptolide induces the same mitochondrial apoptotic pathway leading to cardiotoxicity in cardiomyocytes. It is noteworthy that this process is p53-dependent [133]. Although triptolide affects the expression of Nrf1 and Nrf2 in cardiomyocytes, there are currently no studies that elucidate how triptolide disrupts the reciprocal regulation of Nrf1 and Nrf2 in mitochondrial redox homeostasis. This may prove to be a pivotal step in the development of more efficacious strategies for the reduction of triptolide-induced cardiotoxicity.

### Nephrotoxicity

Another significant limitation of the clinical use of triptolide is nephrotoxicity. In 2019, Malireddi proposed a novel cell-death mode known as PANoptosis, that integrates pyroptosis, apoptosis, and necroptosis through the assembly of PANoptosome complex. The PANoptosome, which is assembled by kinases such as ZBP1, is crucial for the regulation of PANoptosis [134]. A recent study demonstrated that triptolide disrupts mitochondrial function and promotes the assembly of the PANoptosome through the increase of ROS. In vivo experiments demonstrated that both nephrotoxicity and hepatotoxicity of triptolide are highly correlated with the occurrence of the PANoptosome [135]. Another study proved that oxidative stress induced by increased ROS also led to mtDNA damage and leakage, which activated the cGAS-STING pathway, and ultimately leading to nephrotoxicity [136].

### Ototoxicity

Triptolide inhibits the viability and proliferation of inner ear stem cells, thereby causing hearing damage. Triptolide interferes with the oligomerization process of OPA1, leading to the abnormal division of OPA1 and ultimately preventing the mitochondrial fusion of inner ear stem cells [137].

## Mitochondria targeted therapeutic optimization: structural engineering and synergistic combination therapies

### Mitochondria interactions of derivatives and novel dosage forms

In comparison to normal mitochondria, mitochondria in tumor cells exhibit a greater degree of variability. Based on the differences in pH and membrane potential of tumor cell mitochondria, many strategies for targeting tumor cell mitochondria have been devised [138]. It has been reported that celastrol can target MRC complex I [55]. Meanwhile, both celastrol and triptolide can induce mitochondrial apoptosis. Therefore, by modifying the structure of the compounds or the dosage form, it is possible to design new derivatives and dosage forms with enhanced mitochondrial targeting ability (Table 3). These have unique advantages, including improved bioavailability, reduced toxicity, improved water solubility, enhanced targeting and altered modes of delivery.

**Table 3:** Mitochondrial targeted nanodelivery system containing the active compounds of TWHF

Natural product	Platform	Particle size	EE%	LE%	Model	Composition	Key findings	Ref.
Celastrol	Micro-emulsion	40.02 ± 0.21 nm	94.50%	40.97%	HeLa 3D tumor spheroid	Microemulsion, celastrol	A mitochondrial-targeted microemulsion for cervical cancer therapy	[142]
	Polymeric micelle	63.5 ± 18.0 nm	92.34 ± 0.66%	12.18 ± 0.10%	MCF-7, A549, L-02; nude mice	Triphenylphosphonium bromide, glucolipid-like conjugates, celastrol	A micelles with mitochondrial targeting and alkaline pH-responsive release capability for cancer therapy	[144]
	Polymeric micelle	100 nm	–	42 ± 2%	4T1, B16F10, A549	Poly(2-(N-oxide-N,N-dimethylamino)ethyl methacrylate)-block-poly (2-hydroxyethyl methacrylate), celastrol	A high bioavailability complex with dual mitochondrial targeting	[145]
	Polymeric micelle	121.10 ± 11.08 nm	–	15%	A549; nude mice	Amido amine, celastrol, polyethyleneglycol	A GLUT1-targeting and hypoxia-activated mitochondria-targeted complex for cancer therapy	[146]
	Liposome	88.97 ± 1.27 nm	98.37 ± 0.28	–	HepG2 cells; BALB/c-nude mice	Hyaluronic acid, triphenylphosphine, celastrol	A complex with dual targeting of mitochondrial and tumor cell surface receptors	[147]
	Metal–organic framework	234.5 nm	60.52% ± 2.79	31.60% ± 2.85	SKOV3, ES-2, IOSE80	ZIF-8, PEG-BIO, celastrol	A highly water-soluble complex that triggers apoptosis of tumor cells by affecting mPTP	[151]
	Metal–organic framework	–	–	–	AR42J, RAW264.7; CDE mice	ZIF-8, Prussian blue, celastrol	A Metal–Organic framework through ROS scavenging and mitophagy activation to restore mitochondrial homeostasis alleviates severe acute pancreatitis	[153]
	Others	99.65 nm	–	–	MCF-7 tumor-bearing mice	Triphenylphosphine, celastrol, all-trans retinoic acid, cisplatin	A dual-drug multi-stage delivery system with mitochondria- and nucleus-targeting capabilities for synergistic tumor therapy	[148]
Triptolide	Liposome	133 nm	62.67 ± 4.59%	0.76 ± 0.01%	Pan02; C57BL/6 J mice	Amphiphilic stachydrine-octadecane conjugate, polyethylene glycol, liposome surfaces, triptolide	A liposome-based delivery system with mitochondria-targeting capability for enhanced pancreatic cancer therapy	[150]
	Nano-hydrogel	80.87 ± 0.52 nm	83.52%	4.52%	HUVECs, MDA-MB-231, MCF-7, 4T1; BALB/c mice	Poly (N-isopropylacrylamide-co-acrylic acid), p(NIPAAm-co-AAc)-g-F68 copolymer, triptolide	A thermo-sensitive hydrogel that enhances levels of mitochondrial apoptosis and inhibits tumor angiogenesis	[152]
	Polymeric micelle	148 nm	71.10%/	12.45%	HK-2 cells; C57BL/6 J mice, SD rats	triphenylphosphonium, lysozyme, triptolide	A mitochondria-targeted nanoplatform enabling triple-pathway renal accumulation for acute kidney injury therapy	[154]

*EE%* drug encapsulation efficiency, *LE%* drug loading efficiency

The derivatives CK-21 [31] and 10b [139] were created by structural modification of triptolide and celastrol, respectively. They were both found to be more potent in causing mitochondrial dysfunction and inducing apoptosis in tumor cells. Triphenylphosphine (TPP) is capable of crossing cell membranes due to its high lipid solubility. Its high positive charge facilitates its binding to the highly negatively charged mitochondrial membrane of tumor cells. Therefore, TPP is an excellent candidate for mitochondria-targeted structural modifications [140]. Modification of the TPP^+^ moiety at the C-14β-OH site of triptolide screened out the optimal structure, Mito-TP-2, with excellent mitochondrial targeting properties. This derivative was shown to retain its pro-tumor apoptotic effects and exhibited no significant toxicity [141].

In the exploration of nano delivery systems targeting mitochondria, the transferrin-modified microemulsions containing celastrol and coix seed oil (Tf-CT-MEs) enhanced apoptosis in tumor cells by targeting mitochondria, showing a 2.77-fold improvement in tumor suppression compared to direct celastrol treatment [142]. Micelles are amphiphilic molecules that form spherical structures in water, with hydrophobic cores and hydrophilic shells. They encapsulate poorly water-soluble drugs, improving their solubility and delivery. Micelles have shown promise in targeted cancer therapy, particularly in targeting the mitochondria of tumor cells [143]. Three nano micellar delivery systems (OPDMA-HEMA, CTPP-CSOSA/Cela, Glu-PEG-Azo/Mito-Cel808) have been developed for the delivery of celastrol in cancer therapy, all with mitochondrial targeting [144–146]. CTPP-CSOSA/Cela can also release celastrol in response to the mitochondrial pH environment. The tumor inhibition rate of it reached 80%, significantly surpassing that of CSOSA/Cela and celastrol [144]. Glu-PEG-Azo/Mito-Cel808 targets the GLUT1 protein, facilitating faster cellular uptake and inhibiting ATP production in tumor cells compared to the previous two [146]. In conclusion, all three types of micelles loaded with celastrol nanosystems have good mitochondrial targeting and can enhance mitochondrial pathway-induced apoptosis in tumor cells. Liposomes are commonly used in nano delivery systems for targeted drug delivery to improve efficacy and reduce side effects. Using the above mentioned mitochondrial targeting molecular TPP, combined with celastrol to form liposomes. The shell-encapsulated HA to make C-TL/HA has been demonstrated to effectively target mitochondria and promote apoptosis in tumor cells [147]. In another study, two novel pro-drug molecules (TPP-Cet and RA-Pt) were co-loaded into the Fc-INPs. It could target and deliver celastrol and Pt to the mitochondria and nucleus of tumor cells, respectively, to achieve a synergistic therapeutic effect. As a result, the tumor inhibition rate of RA-Pt/TPP-Cet@Fc-INPs reached 81.5% [148]. Choi prepared a celastrol-loaded system (CMSN-PEG) and found it to possess exceptionally high drug-loading efficiency and mitochondrial targeting. CMSN-PEG decreased the GSH/GSSG ratio in the mitochondria and inhibited angiogenesis and tumor cell proliferation through controlled release [149]. Triptolide-liposome conjugated with SS forms SS-TP LPs, which possess mitochondrial targeting properties. Further in vivo and in vitro experiments have shown improved efficacy against pancreatic cancer with minimized side effects [150]. Zeolitic imidazolate framework-8 (ZIF-8) is pH-sensitive and dissolves in the acidic environment of tumors while remaining stable in normal tissues and cells. A innovative drug delivery system (CEL@ZIF-8@PEG-BIO) has been developed with more efficacious than free celastrol, which can significantly disrupt Δψm and release ROS [151]. Another innovative hydrogel drug delivery system for encapsulating triptolide, TPL@nano-gel, possesses the capability of sustained drug release. Compared to free triptolide, TPL@nano-gel not only enhances the mitochondrial apoptosis pathway but also inhibits anti-tumor angiogenesis via VEGFR-2 signaling. Its"second-strike"ability demonstrates its high anti-tumor potential [152]. Recent studies have leveraged the strong anti-inflammatory effects of triptolide/celastrol to design nano-delivery systems for various inflammatory diseases. A macrophage membrane-coated nanosystem (MPBZC) specifically targets pancreatic inflammation to deliver celastrol. This approach restores mitochondrial autophagy and scavenges ROS, significantly improving mitochondrial homeostasis and increasing survival rates in severe acute pancreatitis from 12.5 to 62.5%. The system offers dual therapeutic action against oxidative stress and impaired mitophagy [153]. Another study develops a triple-targeting nanoplatform (TL-N@TP) that enhances triptolide delivery for acute kidney injury therapy through glomerular filtration, renal tubule uptake, and mitochondrial localization, effectively improving renal function while reducing oxidative stress and inflammation [154].

Despite significant advancements in mitochondria-targeted nano-delivery systems for triptolide and celastrol, several limitations persist. While the clinical translation of triptolide and celastrol remains constrained by challenges such as systemic toxicity and poor solubility, significant progress has been made in developing derivatives and mitochondria-targeted delivery systems to overcome these limitations. For instance, the hydroxylated derivative LLDT-8 has advanced to Phase II clinical trials for rheumatoid arthritis, demonstrating enhanced safety profiles and retained therapeutic efficacy [155]. Furthermore, mitochondria-targeted delivery platforms, including Mito-TP-2 [141] and C-TL/HA [156], have achieved site-specific drug release and toxicity mitigation in preclinical models. These innovations hold promise as breakthrough therapies for cancer and other mitochondria-related pathologies.

### Mitochondria mediated synergistic mechanisms in combination therapies

Following the theory of traditional Chinese medicine, some traditional Chinese medicine compounds combined with TWHF can reduce its organ toxicity. Studies have found that the mechanism involves the regulation of mitochondrial function [157]. Therefore, in addition to the strategy of structure optimization and dosage modification, the use of other drugs or compounds combined with triptolide can also significantly improve its multiple organ toxicity. Catapol and Panax notoginseng saponins, two compounds from *Rehmannia glutinosa* and *Panax notoginseng*, have been demonstrated to regulate mitochondrial transcription factor A (TFAM) and Cyt c, which can enhance mitochondrial function and mitigate triptolide-induced hepatotoxicity [158]. Two flavonoids, Calycosin and Hyperoside, have both been reported to improve mitochondrial biogenesis through the PGC-1α pathway, counteracting triptolide-induced cardiac injury and reproductive toxicity [159, 160]. The foregoing suggests that mitochondrial ROS accumulation is a key factor in triptolide-mediated numerous toxicities. MitoQ, as a mitochondria-targeted ROS scavenger that protects mitochondrial function and alleviates oxidative stress, has been demonstrated to protect against cardiotoxic and reproductive toxic levels of triptolide [161, 162]. In addition, ferroptosis due to mitochondrial dysfunction is also involved in the mechanism of triptolide toxicity. Ferrostatin-1, a ferroptosis inhibitor, can reverse triptolide-induced mitochondrial dysfunction and improve reproductive toxicity [163]. These targeted strategies to improve mitochondrial function may also be applicable in treating other organ toxicities induced by triptolide in the future. In summary, investigating the toxic mechanisms of mitochondria in relation to triptolide will facilitate the development of additional detoxification strategies, thereby enhancing the clinical therapeutic potential of triptolide. Additionally, due to the dual role of mitochondria, further studies are needed to assess whether these strategies affect the therapeutic efficacy of triptolide.

## Discussion and future perspectives

The pharmacological mechanisms of TWHF’s principal active ingredients continue to be the focus of ongoing investigation, especially celastrol and triptolide. Additionally, the secrets of their toxicity mechanisms have also been revealed. A mitochondrial-centred mechanistic network involving the mitochondrial biogenesis, mitochondrial fusion-fission dynamics, mitophagy and mitochondrial apoptosis pathway explains the pharmacological and toxicological development of the prime active compounds of TWHF. However, this intricate network requires further elucidation to fully delineate the underlying mechanisms. For instance, Celastrol has been shown to potentiate the mitochondrial apoptotic cascade in tumor cells by upregulating the ΔΨm, thereby exerting therapeutic effects across a spectrum of malignancies [14]. In contrast, in hippocampal neuronal cells, celastrol improved mitochondrial function and inhibited apoptosis by down-regulating 1392-bp noncoding RNA sequent-AK005401 to activate the downstream PI3K/Akt pathway. Finally, it slowed down the neuronal damage [117]. Although the pathological mechanisms ultimately converge on mitochondrial dysfunction, the distinct nature of upstream regulatory targets in normal cellular populations compared to neoplastic cell counterparts may constitute the predominant underlying mechanism driving divergent pathophysiological outcomes. Additionally, the opposing effects of celastrol may be influenced by the tumor microenvironment and its unique mitochondrial homeostasis. The acidic conditions of the tumor microenvironment may promote the accumulation of celastrol. At the same time, the hypoxic environment of tumors activates HIF-α, increasing the likelihood of triggering the mitochondrial apoptosis pathway [164]. Lastly, due to the characteristics of the tumor microenvironment, mitochondrial potential is typically at a critically high level, making it susceptible to perturbations that can induce apoptosis. In contrast, neurons maintain robust ΔΨm regulation through OXPHOS-dependent processes and endogenous ROS buffering systems [165]. To summarize, the characteristics of the tumor microenvironment and the cell type-specific vulnerability of ΔΨm highlight celastrol's dual role as both an oncotherapeutic agent and a neuroprotective candidate. Triptolide exerts its antitumor effects and cardiotoxic properties through p53-dependent mitochondrial pathways, a well-established therapeutic target in oncology. In endometrial cancer, where p53 dysfunction (mutations or inactivation) frequently contributes to therapeutic resistance [166], triptolide demonstrates a unique p53-independent mechanism by directly activating caspase-3/9 through mitochondrial pathway stimulation. This emphasizes the importance of investigating the broad mechanisms by which triptolide regulates mitochondrial functional networks, Its ability to bypass defective p53 signaling in endometrial cancer highlights a potential therapeutic advantage. In addition to the processes of apoptosis, pyroptosis, and the PANoptosome discussed in this paper, it is necessary to determine whether the active ingredients of TWHF mediate the occurrence of other novel modes of cell death by affecting mitochondrial function. This is a subject that requires further investigation by researchers. Many compounds of TWHF have been demonstrated to possess therapeutic potential for inflammatory, metabolic, and neurodegenerative diseases. It should be noted, however, that reports on the protective effects of other compounds on mitochondrial function in the above diseases are relatively limited compared to celastrol. This review shows that the mechanisms of toxicity of the main active components of TWHF partially overlap with the pharmacological mechanisms. Due to the dual roles of mitochondria in toxicological and pharmacological aspects, dosage control and combination strategies for mitigating toxicity may have an impact on the therapeutic efficacy of active ingredients, which require further investigation. It is suggested that a better attenuation strategy is to improve the therapeutic effect of triptolide/celastrol and reduce the accidental damage to normal cells by modifying the dosage form to accurately target the mitochondria at the lesion site. It is acknowledged that certain modifications, such as TPP, may be associated with off-target risks. Future research could focus on three key directions: first, to explore and employing mitochondrial targeting that is more effective or better combined with compounds. Secondly, there is the possibility of further exploration into the potential mitochondrial targeting mechanisms of the active ingredients themselves. For instance, the targeting property of celastrol to mitochondrial VDAC2 [57] can be employed to achieve dual targeting function with modifiers, thereby effectively reducing the risk. Finally, different lesion sites have specific mitochondrial microenvironments that may affect the mitochondrial targeting ability of the system. The development of multi-stage cascade systems that first localize to pathological sites and subsequently deliver payloads to mitochondria could mitigate off-target effects in healthy organs. This approach, supported by recent advances in targeted therapeutics, demonstrates significant translational potential [154]. These approach enables the attainment of the overarching objective of reducing toxicity and enhancing efficacy, with great potential for clinical transformation. In addition to the two most well-known compounds, celastrol and triptolide, further investigation into the mitochondrial effects of TWHF decoction pieces and their other bioactive constituents holds significant promise for broadening their pharmacological applications and advancing their clinical utility.

## Data Availability

Not applicable.

## Abbreviations

AIF: Apoptosis inducing factor
AKT: Protein kinase B
AMPK: Adenosine 5′-monophosphate-activated protein kinase
AT2: Angiotensin II
CDK: Cyclin-dependent kinases
Cyt c: Cytochrome C
Drp1: Dynamin-related protein 1
DUSP1: Dual specificity phosphatase 1
Endo G: Endonuclease G
ERK: Extracellular regulated protein kinases
HSP70: Heatshock protein 70
HSP90: Heatshock protein 90
Mcl-1: Myeloid cell leukemia-1
MRC complex I: Mitochondrial respiratory chain complex I
MMP: Matrix metalloproteinases
mPTP: Mitochondrial permeability transition pore
mTOR: Mammalian target of rapamycin
NF-κB: Nuclear factor kappa-B
Nrf2: Nuclear factor erythroid 2-related factor 2
Nur77: Nerve growthfactor-induced gene B
JNK: C-Jun N-terminal kinase
NSCLC: Non-small-cell lung cancer
OPA1: Optic atrophy protein 1
PARP: Poly ADP-ribose polymerase
PEG: Polyethylene glycol
PERK: Protein kinase R PKR-like endoplasmic reticulum kinase
PGC-1α: Peroxisome proliIerators-activated receptorγcoactivator lalpha
PI3K: Phosphoinositide 3-kinase
RIPK: Receptor-interacting protein kinase 1
PGC-1α: Peroxisome proliferators-activated receptorγcoactivator lalpha
ROCK: Rho-associated protein kinase
ROS: Reactive oxygen species
SIRT1: Sirtuin 1
SS: Stachydrine-octadecane conjugate
TCA: Tricarboxylic acid
TFAM: Mitochondrial transcription factor A
TNBC: Triple-negative breast cancer
TPP: Triphenylphosphine
TRAF: Tumor necrosis factor receptor-associated factor
TWHF: *Tripterygium wilfordii* Hook F
T2DM: Diabetes mellitus type 2
T-96: Demethylzeylasteral
VDAC: Voltage-dependent anion channel
XIAP: X-linked inhibitor of apoptosis protein
ΔΨm: Mitochondrial membrane potential
